# 浊点萃取-气相色谱-质谱法测定水中9种芳香胺类化合物

**DOI:** 10.3724/SP.J.1123.2023.06002

**Published:** 2024-03-08

**Authors:** Chao YANG, Jinglong LIU, Xiaojian XU, Lijuan WU, Mingming YIN, Wei DAI, Qian HAN

**Affiliations:** 1.江苏省南京环境监测中心, 江苏 南京 210013; 1. Nanjing Environmental Monitoring Center of Jiangsu Province, Nanjing 210013, China; 2.江苏省扬州环境监测中心, 江苏 扬州 225007; 2. Yangzhou Environmental Monitoring Center of Jiangsu Province, Yangzhou 225007, China

**Keywords:** 浊点萃取, 气相色谱-质谱法, 芳香胺, 环境水样, cloud point extraction (CPE), gas chromatography-mass spectrometry (GC-MS), aromatic amines, environmental water sample

## Abstract

本研究基于以曲拉通X-114(Triton X-114)为萃取剂的浊点萃取技术和气相色谱-质谱法,建立了一种高效、高灵敏度的水体中9种芳香胺(2-氯胺、3-氯胺、4-氯胺、2-硝基苯胺、3-硝基苯胺、4-硝基苯胺、1-萘胺、2-萘胺和4-氨基联苯)的检测方法。采用单因素优化法对影响提取效果的重要因素进行了优化。采用气相色谱-质谱对水中9种芳香胺进行定性、定量分析,使用中等极性色谱柱DB-35 MS(30 m×0.25 mm×0.25 μm)进行分离,在选择离子模式(SIM)下测定,内标法定量。实验结果表明,9种芳香胺在16 min内能够完全分离,且在各自的范围内线性关系良好,相关系数(*R*^2^)均大于0.998。9种芳香胺的检出限(LOD)和定量限(LOQ)分别为0.12~0.48 μg/L和0.40~1.60 μg/L。选取饮用水源地地表水、近海海水和典型印染行业废水3种类型水体进行加标回收试验,在2个加标水平(2.0、10.0 μg/L)下,饮用水源地地表水的加标回收率为81.1%~109.8%,日内精密度为0.7%~5.2%,日间精密度为1.6%~6.2%;近海海水的加标回收率为83.0%~115.8%,日内精密度为1.5%~8.6%,日间精密度为2.4%~12.2%;典型印染行业废水的加标回收率为91.0%~120.0%,日内精密度为2.9%~12.9%,日间精密度为2.5%~13.1%。采用所建立方法对饮用水源地地表水、近海海水和典型印染行业废水中的9种芳香胺进行检测,在饮用水源地地表水和近海海水中均未检出目标组分,而在印染行业废水中主要检出2-氯胺、4-氯胺和4-氨基联苯3种印染行业常用的芳香胺。与其他方法相比,该方法具有操作简单、灵敏度高、成本低、有机试剂用量少、重复性较好等优点,为水中芳香胺及其他痕量有机物的测定提供了技术支持。

## Abstract (en)

Aromatic amines are a class of compounds bearing amino groups on their benzene rings; these compounds are important raw materials for the industrial production of rubber chemicals, pesticides, dyes, pharmaceuticals, photosensitive chemicals, and agricultural chemicals. Research has revealed that some aromatic amines teratogenetic, carcinogenic, and mutagenic properties. Given the high toxicity and potential harm caused by aromatic amines, monitoring their levels in water sources is critical. Aromatic amines are among the 14 strategic environmental pollutants blacklisted in China, and assessing their exposure levels is essential for protecting human health and the environment. At present, the standard method for detecting aromatic amines in water is liquid-liquid extraction-gas chromatography-mass spectrometry (LLE-GC-MS). However, this method has the disadvantages of large sample size requirement, complex operation, long analysis time, and high reagent consumption. In this study, instead of traditional LLE technology, cloud point extraction (CPE) technology was used in combination with GC-MS to establish an efficient, sensitive, and environment-friendly method for the detection of nine aromatic amines, namely, 2-chloramine, 3-chloramine, 4-chloramine, 2-nitroaniline, 3-nitroaniline, 4-nitroaniline, 1-naphthylamine, 2-naphthylamine, and 4-aminobenzene, in water. Triton X-114 was used as the extraction agent. The main experimental parameters were optimized using a single-factor optimization method. The aromatic amines in various water samples were quantitatively analyzed using GC-MS. The nine aromatic amines were separated on a DB-35 MS capillary column (30 m×0.25 mm×0.25 μm). The mass spectrometer was operated in selected ion monitoring (SIM) mode, and quantitative analysis was performed using the internal standard method. The results demonstrated that all nine aromatic amines could be completely separated within 16 min and had good linearities within accurate mass concentration ranges, with correlation coefficients (*R*^2^) greater than 0.998. The limits of detection (LODs) and quantification (LOQs) of these aromatic amines in water were 0.12-0.48 and 0.40-1.60 μg/L, respectively. The accuracy and precision of the method were assessed via the determination of aromatic amines in surface water of drinking water sources, offshore seawater, wastewater of the typical printing and dyeing industry at levels of 2.0 and 10.0 μg/L. The recoveries of the aromatic amines in surface water of drinking water sources were 81.1%-109.8%, with intra-day and inter-day relative standard deviations (RSDs) of 0.7%-5.2% (*n*=6) and 1.6%-6.2% (*n*=3), respectively. The recoveries of the aromatic amines in offshore seawater were 83.0%-115.8%, with intra-day RSDs (*n*=6) of 1.5%-8.6% and inter-day RSDs (*n*=3) of 2.4%-12.2%. The recoveries of the nine aromatic amines in wastewater of the typical printing and dyeing industry were 91.0%-120.0%, with intra-day RSDs (*n*=6) of 2.9%-12.9% and inter-day RSDs (*n*=3) of 2.5%-13.1%. The established method was used to detect nine aromatic amines in actual water samples. No aromatic amines were detected in the surface water of drinking water sources or offshore seawater samples. However, 2-chloramine, 4-chloramine, and 4-aminobenzene, which are frequently used in the printing and dyeing industry, were detected in the wastewater of the typical printing and dyeing industry samples. The proposed method offers the advantages of simple operation, high sensitivity, low cost, low organic reagent requirement, and good repeatability. Thus, this method provides reliable technical support for studying the residual status and environmental behavior of aromatic amines in water.

芳香胺是一类重要的化工原料,其广泛应用于橡胶化学品、农药、染料、药品、照相化学品的制造,并广泛用作农用化学品的合成中间体。大多数芳香胺具有一定的水溶性^[1,2]^,在工业设施排放的废水中检出率较高。研究表明,许多芳香胺会对DNA造成损害,对水生生物有剧毒,并可通过呼吸道、消化道及皮肤黏膜等途径被生物体吸收,从而对血液系统、神经系统造成不可逆的伤害^[3]^。同时,多数芳香胺还具有潜在的致畸、致癌、致突变作用,被欧盟和美国环境保护局列为优先污染物,我国也将芳香胺作为水污染防治的重点污染物之一^[4]^。

水样中芳香胺的含量较低,且存在基质效应,直接进样会导致芳香胺的测定较为困难,结果准确性差,因此在仪器分析之前必须进行提取和预浓缩步骤。液液萃取(LLE)和固相萃取(SPE)是水样最常用的萃取方法,LLE存在有机溶剂用量大、耗时长等缺点;SPE所需溶剂较少,但成本高,对大体积样品的富集性能较差,在一定程度上限制了其应用。国内外对于水中芳香胺的分析方法主要有分光光度法^[5,6]^、气相色谱法^[7]^、液相色谱法^[8⇓-10]^、气相色谱-质谱法^[11⇓-13]^、液相色谱-质谱法^[14,15]^、毛细管电泳法^[16]^、离子色谱-安培检测法^[17]^、电化学法^[18]^等。以上方法多采用常规的LLE或SPE方式,存在回收率低、测定化合物种类少、样品及试剂用量大等缺点。浊点萃取(CPE)是一种新型的LLE技术,其利用表面活性剂的增溶特性和浊点分相来实现目标物的富集和分离,具有成本低、操作简单、富集系数高、表面活性剂用量少等优点,符合绿色化学的要求。CPE是Watanabe等^[19]^在1978年首次引入分析化学领域、并成功取代常规有机试剂的提取技术,目前CPE已成功应用于金属离子含量及其形态分析、痕量有机物的分离等方面^[20⇓-22]^。

针对上述问题,本文建立了浊点萃取-气相色谱-质谱同时浓缩、分离、测定水体中9种芳香胺类化合物的分析方法,并验证了该方法在不同水体中的适用性,为水体中芳香胺的提取与富集提供了新的研究方向和技术支持。

## 1 实验部分

### 1.1 仪器、试剂与材料

7890B-5977A气相色谱-质谱仪,配有电子轰击电离(EI)源(美国Agilent公司); TGL16M高速冷冻离心机(长沙湘智离心机仪器有限公司); HH-4数显恒温水浴锅(国华仪器制造有限公司); XW-80A涡旋混合仪(海门市其林贝尔仪器制造有限公司); Milli-Q纯水仪(美国Millipore公司)。

乙腈、甲苯、正戊烷、乙酸乙酯、甲醇(色谱纯,美国Merck公司); *N*,*N*'-二甲基甲酰胺、NaCl、Na_2_SO_4_、Na_2_SO_3_、Na_2_S_2_O_3_、NaOH(分析纯)、吐温80、十二烷基硫酸钠(化学纯)(国药集团化学试剂有限公司);曲拉通X-114(Triton X-114,试剂级,上海麦克林生化科技股份有限公司)。

9种芳香胺类化合物及3种内标:2-氯胺(纯度99.1%)、3-氯胺(纯度99.3%)、4-氯胺(纯度99.4%)、2-硝基苯胺(纯度98.5%)、3-硝基苯胺(纯度99.5%)、4-硝基苯胺(纯度98.8%)、1-萘胺(纯度98.8%)、2-萘胺(纯度98.1%)、4-氨基联苯(纯度98.7%)、4-硝基苯胺-d_4_(纯度97.7%)、2-萘胺-d_7_(纯度97.5%)均购自德国Dr. Ehrenstorfer公司;4-氯胺-d_2_(纯度98.6%,加拿大CDN ISOTopes公司)。

### 1.2 标准溶液的配制

用甲醇将9种芳香胺类化合物和3种内标分别配制成1000 mg/L的9种芳香胺混合标准储备液和1000 mg/L的3种内标混合标准储备液,并于-20 ℃冷冻保存。用甲醇进一步将上述两种溶液稀释成质量浓度为10 mg/L的9种芳香胺混合使用液和1 mg/L的3种内标混合使用液。

移取适量的9种芳香胺混合使用液和3种内标混合使用液,用乙酸乙酯稀释成系列质量浓度(5、10、20、50、100、500、1000 μg/L)的混合标准溶液,其中内标的质量浓度均为50 μg/L,备用。

### 1.3 样品的萃取

量取10 mL水样于离心管中,依次加入1 mL Triton X-114水溶液(使得样品中Triton X-114的终浓度为1.0%(质量分数))、1 mL Britton-Robison缓冲溶液(0.04 mol/L H_3_PO_4_-0.04 mol/L HAc-0.04 mol/L H_3_BO_3_-0.2 mol/L NaOH, pH≈11)和0.3 g Na_2_SO_3_,混合均匀后加入20 μL 3种内标混合使用液;将离心管置于水浴锅中,平衡温度设为55 ℃,平衡时间设为15 min,然后在3500 r/min下离心5 min;将离心管置于冰浴中静置5 min,移除水相部分,保留表面活性剂相,加入0.4 mL乙酸乙酯,涡旋混合30 s,取有机相待测。

### 1.4 GC-MS分析条件

色谱柱:Agilent DB-35 MS毛细管柱(30 m×0.25 mm×0.25 μm)。柱升温程序:80 ℃保持1 min;以10 ℃/min升温至160 ℃;以15 ℃/min升温至220 ℃,保持2 min;再以30 ℃/min升温至270 ℃。进样口温度:250 ℃;分流比:5:1;载气:高纯氦气(纯度大于99.999%);柱流量:1.0 mL/min;进样量:1 μL。

离子源:EI源;离子化能量:70 eV;传输线温度:280 ℃;离子源温度:300 ℃;接口温度:300 ℃;溶剂延迟5 min;数据采集模式:选择离子监测(SIM)模式。9种芳香胺类化合物及3种内标的质谱参数见表1。

**表 1:** 9种芳香胺类化合物及3种内标的保留时间和质谱参数

Compound	Retention time/min	Quantitative ion (m/z)	Qualitative ions (m/z)	Internal standard
2-Chloroaniline (2-氯胺)	7.122	127	129, 65	4-chloroaniline-d_2_
3-Chloroaniline (3-氯胺)	8.439	127	129, 65	4-chloroaniline-d_2_
4-Chloroaniline (4-氯胺)	8.483	127	129, 65	4-chloroaniline-d_2_
2-Nitroaniline (2-硝基苯胺)	11.561	138	92, 65	4-nitroaniline-d_4_
3-Nitroaniline (3-硝基苯胺)	12.535	138	92, 65	4-nitroaniline-d_4_
1-Naphthalenamine (1-萘胺)	12.839	143	115, 116	2-naphthalenamine-d_7_
2-Naphthalenamine (2-萘胺)	13.006	143	115, 116	2-naphthalenamine-d_7_
4-Nitroaniline (4-硝基苯胺)	13.964	138	105	4-nitroaniline-d_4_
4-Phenylaniline (4-氨基苯胺)	15.081	169	115, 168	2-naphthalenamine-d_7_
4-Chloroaniline-d_2_(4-氯胺-d_2_)	8.483	131	67	/
2-Naphthalenamine-d_7_(2-萘胺-d_7_)	13.006	150	122	/
4-Nitroaniline-d_4_(4-硝基苯胺-d_4_)	13.964	142	112	/

## 2 结果与讨论

### 2.1 萃取机理的探究

Triton X-114结构中的聚氧乙烯长链可构成亲水基,当水样中Triton X-114的质量分数为1.0%时,其浓度超过临界胶束浓度(CMC),此时会形成亲水基团向外张开成簇而疏水基团向内聚集成核的胶束,从而使芳香胺类化合物能够吸附或增溶于Triton X-114胶束表面定向排列的聚氧乙烯链中。在水相中存在无机盐的情况下,随着水相温度逐渐高于浊点温度(CPT),水相逐渐浑浊,这是因为胶束亲水基在较高温度及无机盐存在的条件下发生了脱水。胶束中的聚氧乙烯链在高温时因脱水而相互吸引,导致胶束表面极性基团占据的有效面积减小,胶束尺寸增大,引起相分离,从而得到富含芳香胺的表面活性剂相和含少量表面活性剂单体的水相,以此实现芳香胺类化合物的富集和分离。

### 2.2 浊点萃取条件的优化

#### 2.2.1 表面活性剂的选择

表面活性剂的种类是影响CPE萃取效率的重要因素,实验选取了3种不同类型的表面活性剂(十二烷基硫酸钠、吐温80和Triton X-114),分别考察三者对水样中9种芳香胺类化合物的提取效果。实验发现,随着温度升高至80 ℃,加入十二烷基硫酸钠和吐温80的水样始终保持澄清,而加入Triton X-114的水样在水温超过30 ℃时开始出现浑浊。这是因为十二烷基硫酸钠和吐温80的CPT较高,而Triton X-114的CPT为23~26 ℃,较低的CPT能在一定程度上减少实验过程中由温度过高造成的目标物损失,同时也有利于水相与表面活性剂相的分离。因此后续实验选用Triton X-114作为CPE的表面活性剂。

#### 2.2.2 表面活性剂的质量分数

表面活性剂的质量分数不仅会影响萃取效率,而且还会影响相分离后表面活性剂相的体积^[23]^,表面活性剂的质量分数越小,则离心后表面活性剂相的体积越小,富集倍数越大;但过小的表面活性剂相体积(<20 μL)会导致水相与表面活性剂相分离困难,影响方法的准确性和再现性^[24]^。实验考察了样品中不同质量分数(0.1%~3.0%)的Triton X-114对9种芳香胺类化合物提取效率的影响。结果如图1a所示,随着Triton X-114质量分数的增大,9种目标化合物的回收率先增大后减小,在质量分数为1.0%时,除4-氨基联苯外,其余8种目标化合物的回收率达到最大。这是因为Triton X-114在其质量分数较低时所形成的胶束相对较小,对水相中目标化合物的增溶效果较差,萃取回收率偏低;而当Triton X-114的质量分数大于1.5%时,表面活性剂相的体积较大,表面活性剂分子因相互碰撞易聚集成液滴,导致分散不均匀,萃取效率急剧下降。因此,确定样品中Triton X-114的质量分数为1.0%。

**图 1:**
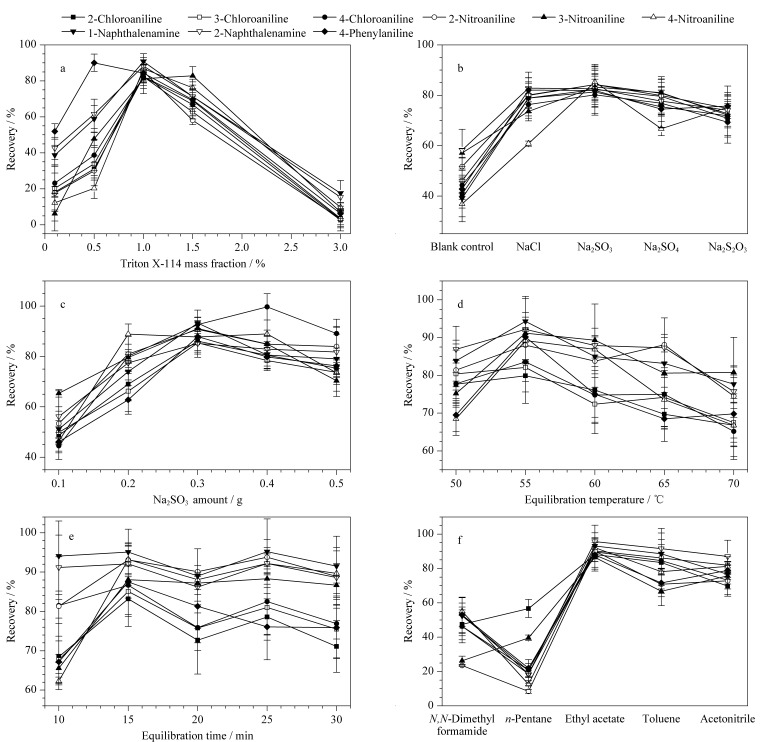
(a)Triton X-114质量分数、(b)无机盐种类、(c)Na_2_SO_3_用量、(d)平衡温度、(e)平衡时间、(f)反萃取剂种类对9种芳香胺类化合物提取效率的影响(*n*=5)

#### 2.2.3 缓冲溶液pH

芳香胺苯环上的氮原子存在孤对电子,在酸性条件下易生成水溶性强的胺盐,导致提取效率下降,因此实验主要考察了中性和碱性条件下目标化合物的提取效率。考虑到实际水样pH的不确定性,采用Britton-Robison缓冲溶液来调节水样pH。考察了不同pH(7、9、11)的Britton-Robison缓冲溶液对9种目标化合物提取效率的影响。结果表明,当Britton-Robison缓冲溶液的pH为11时,9种目标化合物的峰面积最大。萃取剂Triton X-114的结构和性质均较为稳定,基本不受pH波动的影响,因此最终确定Britton-Robison缓冲溶液的pH为11。

#### 2.2.4 无机盐的种类及用量

通常在CMC保持不变的情况下,向试样中加入无机盐可以通过盐析效应及增加表面活性剂所形成的胶束尺寸来提高萃取效率,并能在一定程度上降低CPT,促进相分离^[25]^。考虑到芳香胺类化合物易氧化的特点,实验选取了4种常用的无机盐NaCl、Na_2_SO_3_、Na_2_SO_4_、Na_2_S_2_O_3_,考察它们对芳香胺类化合物的抗氧化效果。结果如图1b所示,与空白对照组相比,4种无机盐的加入均提高了目标物的萃取效率,但Na_2_SO_3_的萃取效果最好,因此后续选择Na_2_SO_3_进一步研究无机盐用量对CPE萃取效率的影响。实验考察了0.1~0.5 g Na_2_SO_3_对9种芳香胺类化合物回收率的影响,结果如图1c所示,当Na_2_SO_3_的加入量为0.3 g时,有8种目标化合物的回收率达到最大。实验结果说明,添加合适的无机盐能够通过盐析效应来降低芳香胺类化合物在水相中的溶解度,并降低表面活性剂相中水的含量,使目标化合物的回收率随无机盐用量的增加而增大。当实验中的Na_2_SO_3_用量过高时,表面活性剂相会变成乳白色,并形成黏性液晶相,难以进行相分离。因此,最终选择0.3 g Na_2_SO_3_进行后续实验。

#### 2.2.5 平衡温度和平衡时间

Triton X-114的CPT为23~26 ℃,理论上CPE最合适的平衡温度应比CPT高15~20 ℃^[26]^,实验考察了不同平衡温度(50~70 ℃)对9种芳香胺类化合物萃取效率的影响。结果如图1d所示,随着平衡温度从50 ℃增加到55 ℃,目标物的回收率也随之增大;继续升高平衡温度,目标物的回收率出现了不同程度的下降,这是因为随着平衡温度的升高,Triton X-114结构中的氢键被破坏,从而引起胶束脱水,水相与表面活性剂相混合不均。因此,选择在55 ℃下进行平衡时间的优化。实验进一步考察了不同平衡时间(10~30 min)对9种目标化合物萃取效率的影响,从图1e中可以观察到,当平衡时间从10 min增加到15 min时,大多数目标化合物的回收率呈上升趋势,继续延长平衡时间,9种目标化合物的回收率保持不变或有所下降。因此,最终确定平衡温度为55 ℃,平衡时间为15 min。

#### 2.2.6 反萃取剂的选择

表面活性剂具有低挥发和高黏度的特性,不能直接进入气相色谱-质谱仪,为了避免表面活性剂对进样系统及质谱造成污染,需采用反萃取剂将表面活性剂相中的目标物萃取至有机相中,从而降低溶液黏度。分别选取*N*,*N*-二甲基甲酰胺、乙腈、甲苯、正戊烷、乙酸乙酯5种不同极性的溶剂作为反萃取剂,考察5种反萃取剂对9种芳香胺类化合物反萃取效果的影响。结果如图1f所示,当乙酸乙酯作为反萃取剂时,萃取效果最优,其次是甲苯和乙腈,*N*,*N*-二甲基甲酰胺和正戊烷的萃取效果明显不如上述3种反萃取剂,其中正戊烷的萃取效果最差。分析其原因,9种目标物均具有一定的极性,因此极性较低的正戊烷萃取效果较差;*N*,*N*-二甲基甲酰胺与Triton X-114混合时会产生白色絮状沉淀,易对目标物产生吸附,造成萃取效率下降;乙腈、甲苯和乙酸乙酯也具有一定的极性,其中乙酸乙酯中的氢键受体最多,能够与目标物形成更强的氢键作用,有利于获得较高的萃取效率。因此,实验选择乙酸乙酯作为反萃取剂。

### 2.3 方法学考察

#### 2.3.1 线性范围、检出限及定量限

对不同质量浓度的混合标准溶液进行测试,以各目标物与内标的峰面积之比为纵坐标(*y*),对应的质量浓度之比为横坐标(*x*),采用线性最小二乘法建立标准曲线。9种芳香胺类化合物在各自的线性范围内均呈良好的线性关系,相关系数(*R*^2^)均大于0.998。以信噪比(*S/N*)分别为3和10时的样品质量浓度作为方法的检出限(LOD)和定量限(LOQ), 9种芳香胺类化合物的LOD为0.12~0.48 μg/L, LOQ为0.40~1.60 μg/L,结果见表2。

**表 2:** 9种芳香胺类化合物的线性范围、线性方程、*R*^2^、LOD及LOQ

Compound	Linear range/(μg/L)	Linear equation	R^2^	LOD/(μg/L)	LOQ/(μg/L)
2-Chloroaniline	5-1000	y=0.952x+0.205	0.9987	0.15	0.50
3-Chloroaniline	5-1000	y=0.771x-0.049	0.9998	0.18	0.60
4-Chloroaniline	5-1000	y=0.673x+0.077	0.9982	0.17	0.57
2-Nitroaniline	10-1000	y=1.872x+0.342	0.9991	0.43	1.43
3-Nitroaniline	10-1000	y=1.306x-0.094	0.9988	0.48	1.60
4-Nitroaniline	10-1000	y=1.163x-0.013	0.9984	0.47	1.57
1-Naphthalenamine	5-1000	y=0.749x+0.270	0.9996	0.12	0.40
2-Naphthalenamine	5-1000	y=1.661x+0.193	0.9998	0.13	0.43
4-Phenylaniline	10-1000	y=0.738x+0.126	0.9996	0.22	0.73

*y*: peak area ratio of the target to the internal standard; *x*: mass concentration ratio of the target to the internal standard.

#### 2.3.2 正确度和精密度

依据《环境监测分析方法标准制订技术导则》(HJ 168-2020)^[27]^对正确度和精密度的规定,选择饮用水源地地表水、近海海水和典型印染行业废水3种实际水样进行加标回收试验,每种水样设置2个加标水平(2.0、10.0 μg/L),每个加标水平平行测定6次,计算方法的加标回收率;一天内测定6次,计算日内精密度;连续测定3天,计算日间精密度,结果见表3。9种芳香胺类化合物的加标回收率为81.1%~120.0%,日内精密度为0.7%~12.9%(*n*=6),日间精密度为1.6%~13.1%(*n*=3),结果表明本方法具有良好的正确度和精密度。

**表 3:** 9种芳香胺类化合物在3种水样中的加标回收率及日内、日间精密度(*n*=6)

Compound	Spiked level/ (μg/L)	Surface water of drinking water sources				Offshore seawater				Wastewater of the typical printing and dyeing industry		
		Recovery/ %	Intra-day RSD/%	Inter-day RSD^*^/%		Recovery/ %	Intra-day RSD/%	Inter-day RSD^*^/%		Recovery/ %	Intra-day RSD/%	Inter-day RSD^*^/%
2-Chloroaniline	2.0	81.6	5.2	4.8		89.2	5.5	4.1		97.2	4.2	5.7
	10.0	92.0	3.1	4.7		107.9	1.5	6.4		91.0	4.7	5.2
3-Chloroaniline	2.0	83.0	3.2	4.8		95.6	3.2	3.5		106.4	7.5	6.6
	10.0	103.9	2.1	2.6		115.8	3.7	4.9		118.2	7.7	11.3
4-Chloroaniline	2.0	81.1	2.2	2.5		90.1	4.4	5.5		105.8	5.6	4.9
	10.0	109.8	1.9	3.3		113.0	2.0	2.7		120.0	4.8	5.3
2-Nitroaniline	2.0	84.9	0.7	1.6		95.2	7.2	6.4		104.1	12.3	13.1
	10.0	104.8	4.7	4.7		103.1	2.7	4.7		109.6	5.1	7.5
3-Nitroaniline	2.0	83.4	4.5	6.2		85.6	8.6	12.2		111.5	8.5	8.1
	10.0	93.4	3.8	3.5		83.0	4.2	5.5		104.2	3.0	4.0
4-Nitroaniline	2.0	83.1	2.7	3.3		85.5	8.1	10.4		114.4	12.9	11.1
	10.0	97.0	1.2	1.8		94.0	3.6	8.6		117.3	2.9	2.5
1-Naphthalenamine	2.0	93.6	1.5	2.9		94.2	2.0	3.8		99.1	9.4	10.3
	10.0	102.8	2.1	2.8		103.5	3.1	2.6		111.6	3.1	3.6
2-Naphthalenamine	2.0	92.7	2.2	3.5		95.1	4.2	11.6		105.6	9.5	7.7
	10.0	89.6	3.5	6.1		86.2	3.3	2.4		98.5	4.3	5.1
4-Phenylaniline	2.0	95.9	3.3	5.0		92.3	5.1	4.7		117.3	4.6	5.2
	10.0	91.4	1.0	3.5		91.5	4.0	7.3		99.0	4.8	2.7

* *n*=3.

#### 2.3.3 方法比较

将本方法与已报道文献作比较,结果见表4。与常用的LLE和SPE相比,本方法的样品取样体积更小,仅为10 mL;与分散液液微萃取法(DLLME)相比,虽然实验所需的样品体积略大,但本方法在芳香胺的检测种类上远多于文献报道^[9,13]^,具有显著优势。LOD是评价方法灵敏度的重要指标之一,本方法的LOD低于多数文献方法,虽有文献方法的LOD比本文更低^[11]^,但其所需的样品体积和富集倍数远超本方法。在回收率方面,本方法中9种芳香胺类化合物的加标回收率范围略宽于其他方法,这可能是复杂水体的基质效应所致。

**表 4:** 水中芳香胺类化合物的分析方法比较

Method	Sample volume/ mL	LODs/(μg/L)									Recoveries/ %	Ref.
		2-Chloro- aniline	3-Chloro- aniline	4-Chloro- aniline	2-Nitro- aniline	3-Nitro- aniline	4-Nitro- aniline	1-Naphth- alenamine	2-Naphth- alenamine	4-Phenyl- aniline		
CPE-GC-MS	10	0.15	0.18	0.17	0.43	0.48	0.47	0.12	0.13	0.22	81.1-120.0	this work
CPE-UV-Vis	10	/	/	/	50	80	60	/	/	/	98.6-115.0	[5]
CPE-LC	15	/	/	/	2	5	13	/	/	/	80.5-109.3	[8]
DLLME-LC	2	/	/	/	0.3	/	0.5	/	/	/	87.6-101.8	[9]
SPE-LC	1000	/	/	/	/	/	0.5	/	/	/	72.2-96.1	[10]
LLE-GC-MS	1000	0.045	0.022	0.035	0.013	0.046	0.035	/	/	/	80.44-98.79	[11]
DLLME-LLE-GC-MS	150	/	/	0.05	/	/	/	/	0.05	/	75.6-115.1	[12]
DLLME-GC-MS	5	0.3	/	/	1.5	/	/	/	/	/	94.7-106	[13]

CPE: cloud point extraction; DLLME: dispersive liquid-liquid microextraction; LLE: liquid-liquid extraction; /: not mentioned.

#### 2.3.4 实际样品检测

将本文所建立的方法应用于饮用水源地地表水、近海海水和典型印染行业废水3种实际水样的检测,每个样品均重复测定3次。结果表明,在饮用水源地地表水和近海海水中均未检出目标组分,而印染行业废水中主要检出2-氯胺、4-氯胺、4-氨基联苯,其质量浓度分别为119.2、100.8、62.9 μg/L, RSD分别为3.3%、2.1%、3.9%,检出组分与印染工艺过程中存在的芳香胺种类相对吻合,加标前后印染行业废水样品的色谱图见图2。实验结果说明,本文所建立方法能够用于水体中芳香胺类化合物的检测及健康风险评估。

**图 2:**
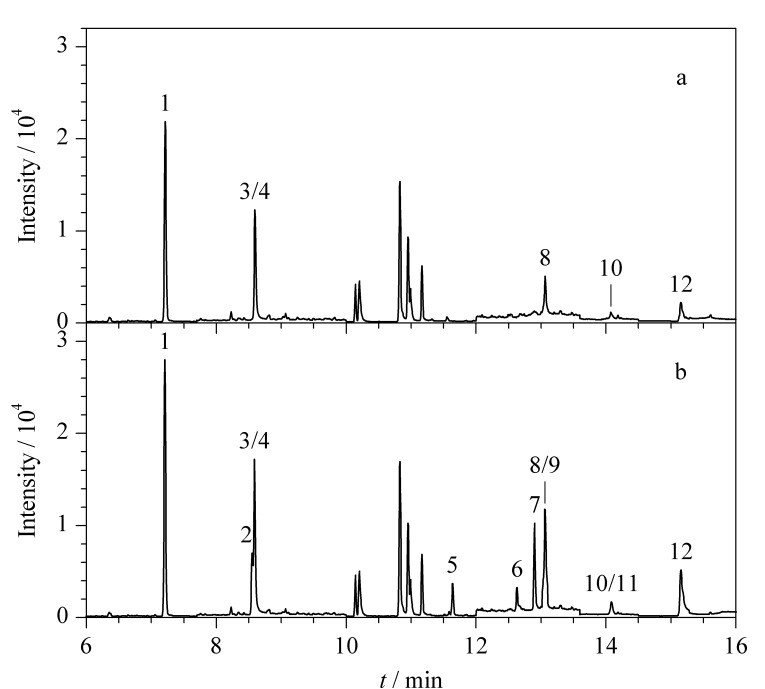
印染行业废水(a)加标前和(b)加标(100 μg/L)后的色谱图

## 3 结论

本文建立了以Triton X-114为萃取剂的浊点萃取方法,并结合气相色谱-质谱法测定了水体中的9种芳香胺类化合物。实验通过反萃取将表面活性剂相中的目标物萃取至有机溶剂中以降低溶液黏度,避免进样系统的污染。与其他常用前处理方法相比,本方法操作简单,灵敏度高,成本低,符合绿色化学的要求;但所采用的浊点萃取技术仍存在不足之处,如富集后的表面活性剂相黏度较高且具有较强的紫外吸收,给直接进样带来不便,并在一定程度上限制了其在液相色谱及液相色谱-质谱仪器中的应用;而采用稀释或反萃取的方式降低黏度又不利于分配系数和富集因子的提高。因此,后续研究将继续探索其他类型表面活性剂在有机物浊点萃取方面的应用,并进一步拓展方法适用的样品类型,为研究有机污染物在环境介质中的污染水平提供方法依据。
